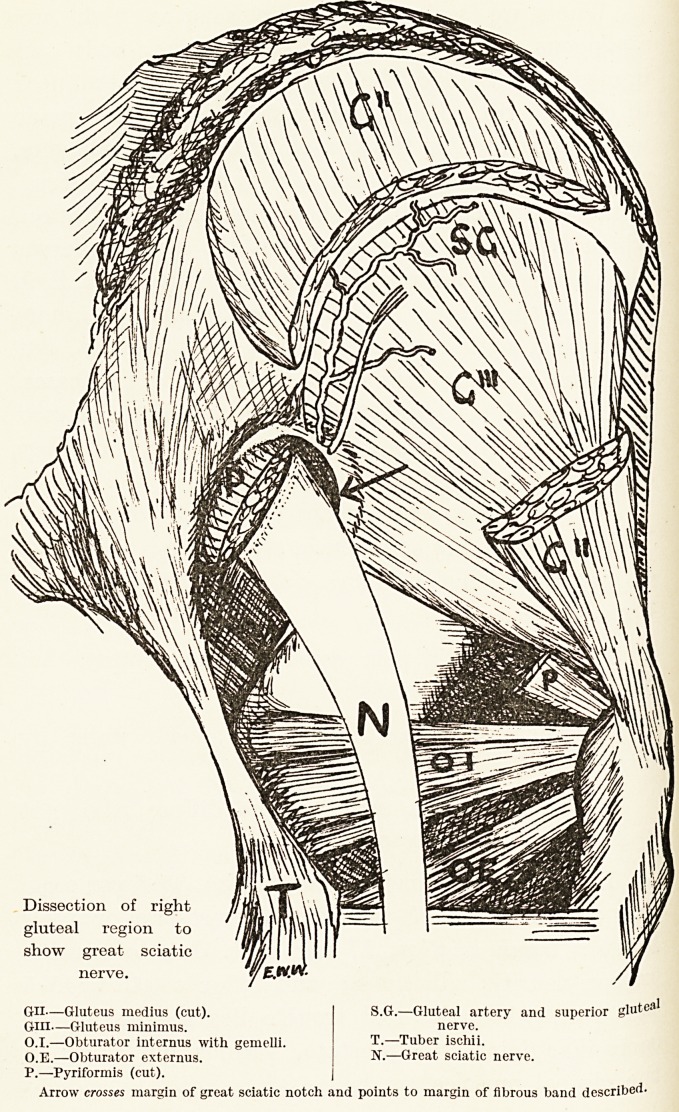# A Treatment for Sciatica
*A Paper read before the Bristol Medico-Chirurgical Society on Wednesday, 8th April, 1936.


**Published:** 1936

**Authors:** A. Rendle Short

**Affiliations:** Surgeon, Bristol Royal Infirmary; Professor of Surgery, University of Bristol


### A TREATMENT FOR SCIATICA.*

BY

A. Rendle Short, M.D., B.S., B.Sc., F.R.C.S.,

*Surgeon, Bristol Royal Infirmary;*
*Professor of Surgery, University of Bristol.*

---

This communication does not attempt to deal at all fully with all the treatments that have been used for sciatica. Alterations in diet, cushions on chairs, hydrotherapy, massage, diathermy, various forms of electrical treatment, bed and a long Liston splint, the application of blistering plasters, the painting on the thigh of strong hydrochloric acid, injections into the nerve or into the sacral hiatus, stretching the nerve or freeing it of adhesions, and doubtless many other therapeutic devices, have been used with more or less success, and I have cured sciatica by removing tumours from the pelvis. I shall have nothing further to say about any of these. Sciatica, of course, is only a name for a group of symptoms, and before it can be treated rationally one must know what is the underlying cause.

It is scarcely necessary to refer at any length to the well-known signs and symptoms of this most troublesome complaint, except to indicate those

* A Paper read before the Bristol Medico-Chirurgical Society on Wednesday, 8th April, 1936.

features that may throw a light upon its nature. Seven signs are given by Purser, viz. pain, Valleix's tender points, Lasègue's sign, absence of ankle-jerks, muscular wasting and weakness, and impaired cutaneous sensation. The principal source of annoyance, of course, is a deep boring or aching pain in the buttock and down the back of the thigh and the leg, usually made worse by movement, or by anything that ever so lightly puts the sciatic nerve on the stretch. If the pain extends below the knee, it is more often met with in the distribution of the external than of the internal popliteal nerve.

Sciatica is seldom bilateral ; if it is the cause will probably be some definite disease such as a pelvic tumour or a growth of the spine or spinal cord. Sciatica may be associated with lumbago.

The physical signs are sometimes, especially in early cases, absolutely negative, but as a rule one can elicit three, viz. tenderness on deep pressure on the buttock over the point of emergence of the nerve through the sacro-sciatic notch, tenderness along the nerve trunk down the back of the thigh and over the external popliteal nerve where it crosses the fibula, and Lasègue's sign, that is, pain when the knee is extended with the thigh flexed. In certain cases there is a slight lumbar scoliosis, the convexity of the curve being towards the painful side. When a severe sciatica has persisted for many months the ankle-jerks may be lost or diminished, and there may be wasting and weakness of the thigh, and especially of the muscles of the leg supplied by the external popliteal nerve. The knee-jerk may be exaggerated or reduced. Occasionally sensation is impaired.

When we seek to compare sciatica with any analogous condition elsewhere in the body we immediately think of the train of symptoms attributed to cervical rib. The long-persistent pain, often made worse by stretching the arm or by certain movements, and the muscular wasting, are very similar. True, some cases of cervical rib show numbing of sensation, which is unusual in sciatica, but the sensory nerves play a much more important part in the upper limb than in the lower. Vascular symptoms are more often seen with cervical rib than with sciatica, but they are only present in a minority of the cases. Medical and electrical treatment sometimes succeed in relieving the pain of both conditions, but they sometimes fail.

No one cause will explain every case of sciatica. We may, with fair confidence, separate out a number of groups. Mills Renton[1] classifies the cases into three types :—

1. Those with pain severe on movement, but entirely relieved by rest.

2. Those with continuous pain, made worse by movement.

3. Those with a rather vague diffuse pain, not aggravated by movement.

J. B. Burt[2] classifies as *root sciatica*, 35·5 per cent. due to affections of the lumbar vertebræ or their joints; *trunk sciatica*, 17 per cent. due to neuritis or peri-neuritic adhesions; and reflex sciatica, 45·5 per cent. when the real trouble lies in the hip or sacro-iliac joint, the prostate, various bursæ, or in the gluteal muscles.

Dissection of right gluteal region to show great sciatic nerve.

GII.—Gluteus medius (cut).

GIII.—Gluteus minimus.

O.I.—Obturator internus with gemelli.

O.E.—Obturator externus.

P.—Pyriformis (cut).

S.G.—Gluteal artery and superior gluteal nerve.
T.—Tuber ischii.
N.—Great sciatic nerve.

Arrow *crosses* margin of great sciatic notch and points to margin of fibrous band described.

I prefer to classify as follows :—

1. *The fibrositis group.* When pain in the sciatic region is associated with lumbago, is vaguely distributed, and yields quickly to medical treatment and local applications of heat or counter-irritants, there is no scoliosis, and no muscular wasting, or loss of reflexes, it may be classified as belonging to this group, fortunately quite a considerable proportion of the whole.

2. *Pelvic growths.* It is quite unusual for sciatica to be the first symptom of cancer of the rectum, but I have seen one case, in a medical man who had noticed no irregularity of bowel action and no bleeding. The sciatic pain was due to metastatic glandular involvement closely adherent to the sacrum near a neural foramen. The carcinoma was diagnosed by a rectal examination. Other pelvic growths, both in men and women, may give rise to pain down the back of the leg. Rectal and vaginal examinations are therefore essential, but very few of our cases coming up as sciatica fall into this group. Scybalous masses in the rectum may cause, or aggravate, sciatica.

3. *Growths of the spine, or spinal cord.* This group is smaller still ; the pain is likely to be bilateral, and other signs or symptoms will surely be present.

4. *Sciatica with lumbar scoliosis.* Putti[3] has demonstrated that there is a group of cases of inveterate sciatica due to osteo-arthritis of the intervertebral articulations, which may be visible in a good skiagram taken specially to show these little joints. In a typical case the arthritis will be on the same side as the pain, rigidity of the lumbar spine is present, and the patient will curve the spine laterally so as to relieve pressure on that side, that is to say the convexity will be to the side of the sciatica. Ordinary medical treatment is useless, but most of these cases can be rid of their pain by prolonged fixation in plaster, at first making no attempt to correct the scoliosis.

5. *Sacro-iliac disease.* Pain down the sciatic nerve is a regular symptom in sacro-iliac tuberculosis, and may be met with also in sacro-iliac strain. Lasègue's sign will probably be present in both conditions. The tenderness of sacro-iliac disease is over the joint, not over the sciatic nerve, and other signs will usually be present if looked for. A. H. Freiberg and T. H. Vinke[4] suggest that the sciatic pain may be due to pressure from spasm of the pyriformis muscle, or to adhesions between the muscles and the nerve.

6. *The remainder.* When we have accounted for all the cases of sciatica that fall into the above groups a large proportion remain on our hands, and amongst them many of the severest, and the most resistant to treatment. Numerous suggestions have been made as to the pathology. It is very likely correct to attribute some of them, especially when there is marked tenderness of the nerve trunk, wasting, and loss of reflexes, to neuritis of the sciatic nerve. The neuritis may presumably be due to some chemical or bacterial poison. Actual proof, histological or otherwise, of the presence of a neuritis in sciatica is almost totally wanting, and several writers, including Hauser[5] and Purser,[6] doubt if it really occurs. Unfortunately, also, this diagnosis does not point the way to a quick and easy method of treatment. An attempt to find out and cut off the source of the poison is indicated, but one has to proceed by the method of trial and error, and the cure is tedious and uncertain. Nerve stretching has been recommended, but it is difficult to see how it can assist matters ; it often fails, and when it succeeds the benefit may be otherwise explained. O. Wiedhoff[7] found that Lasègue's sign is abolished by sacral anæsthesia, but not, in eight cases, by anæsthetic blocking of the nerve trunk. This suggests that the cause of pain lies high up in the nerve. Various deformations of the lumbo-sacral articulation, or of the neural foramina, have been blamed for certain cases of sciatica, and skiagraphic evidence produced to demonstrate them. More often X-rays throw no light on the problem, nor is it easy to see what useful treatment may be indicated if bony deformity is suspected. According to Halweg,[8] of Copenhagen, who has handled 750 cases, the trouble in many cases lies in the muscles of the back of the thigh, not in the nerve trunk at all. He maintains that harder nodules can be felt here and there in the muscles. More promising is the opinion that sciatica may often be due primarily to neuritic adhesions between the nerve and the structures which surround it at the sacro-sciatic notch. Acting on this belief, surgeons have exposed the nerve and endeavoured to free it from adhesions with the finger. This method of treatment has met with a good deal of success. Crawford Renton,[9] in 1897, described a method of operating in which the nerve is exposed below the gluteus maximus by a four or five inch longitudinal incision, hooking it up, and then carefully removing all adhesions from the sacro-sciatic notch to about the middle of the thigh. The adhesions, he says, may be fine and readily separated by the finger, or they may consist of strong bands that have to be divided.

The branches must, of course, be preserved. No splint is necessary. Patients may be cured by this procedure who were not better after the nerve stretching. As a general rule they are free from pain as soon as they get up, but sometimes a few weeks elapse before the pain completely disappears. Crawford Renton had 32 cases, all successful. Mills Renton[10] adds 10 more, 8 were freed from pain, and 2 were not; these belonged to his second and third types.

### SOME PERSONAL EXPERIENCES.

**Case 1.**—In November, 1934, Miss D. W., aged 27, came under my care with severe pain in left hip and knee for five years, with tenderness along the sciatic nerve trunk. Lasègue's sign positive. One-third of an inch wasting of the leg. No scoliosis; rectal examination negative; skiagram showed no bony abnormality. Had been in bed a month with a splint, relapsed as soon as she got up. Quite unable to work as a cook.

*Operation*, 20th November, 1934. I decided to follow the Rentons' advice and operate with a view to separating the sciatic nerve from adhesions where it emerges from the pelvis. The patient was laid face downwards, the nerve exposed at the upper part of the thigh and followed up to the point of its emergence. It was found necessary to cut the lower fibres of the gluteus to obtain good access. On sweeping the finger round the nerve at its emergence there did not appear to be any adhesions, though some loose tissue was torn through, but on the antero-external aspect, closely applied to the nerve, I felt a sharp crescentic edge of dense fibrous tissue, very tightly stretched. Remembering one's experiences with operations for so-called cervical rib (which will be referred to hereafter), it seemed almost certain that this band had been pressing on the outer side of the nerve. I therefore passed up a scalpel blade along my finger, and notched the crescentic edge to the depth of perhaps a quarter of an inch. There was no bleeding. Next day the patient declared her pain had gone.

She resumed her work soon after leaving hospital, and up to the present (March, 1936) has kept quite free from the old pain without any further treatment.

Two further cases of intractable sciatica have since come under my care. In these I approached the point of exit of the nerve not by Crawford Renton's incision with transection of the fibres of the gluteus maximus, but by splitting that muscle directly over the nerve in the line of its fibres. Again a tense band was felt closely applied to the antero-external side of the sciatic nerve in the same situation as before. The superior gluteal artery was not detected. The band was divided with a blunt hernia knife.

**Case 2.**—Miss L. W., aged 27. Pain from bottom of back and down right leg; six months' history. Worse first thing in the morning. Much treatment, no relief. No tenderness along line of sciatic nerve, no wasting, no anæsthesia. Knee and ankle-jerks normal. Lasègue's sign positive. Cannot sit up with leg extended and knee straight without pain at back of knee. Rectal examination negative; no lumbar scoliosis; skiagram shows no bony abnormality.

*Operation*, 7th June, 1935. Sciatic nerve exposed by incision at back of thigh, found normal. Another incision over exit of nerve in line of fibres of gluteus maximus, fibres separated, nerve picked up. Crescentic edge of fibre band as in Case 1 found and divided.

Next day patient was free of pain. There was a slight return when she got up, and she was given an injection of normal saline by the sacral hiatus. At first this did not relieve, but after a few weeks she reported herself quite well.

**Case 3.**—Miss M. G., aged 26. Pain in the left leg for two years. Pain starts in the buttock and goes down the back of the thigh and leg to the sole of the foot, is present more or less at the time. Has not been relieved by massage and electrical treatment. On examination the point of maximum tenderness was over the tip of the sacrum, there was slight tenderness along the course of the sciatic nerve. Flexion of the hip caused pain in the back, extension of the knee gave pain behind the joint. There was no muscular wasting, no anæsthesia, no alteration of reflexes. The signs were therefore not quite typical of sciatica.

*Operation*, 30th July, 1935. The nerve was approached by splitting fibres of the gluteus maximus. The fibrous band was found and divided. In this case no relief followed, but since the end of 1935 I am told she has been free from pain.

The clue to the treatment adopted in my first case was my recollection of experience in operating for what is called "cervical rib." It has long been recognized that the nerve-pressure symptoms attributed to this deformity are, as a matter of fact, much more frequently met with when a genuine rib, articulated to the seventh cervical vertebra, is absent than when it is present. What is often called "cervical rib" is merely an enlargement of the transverse process, and it may be difficult to demonstrate at operation that this particular piece of bone is, as a matter of fact, in close contact with any cord of the brachial plexus. In 1919 Stopford[11] advanced evidence that the real source of the pressure may be the normal first rib, and rasping off the scalene muscles together with removal of a short length of the rib, thus breaking its continuity, relieves the pain. W. M. Brickner,[12] in 1927, agreed in principle with this conclusion.

In 1927 A. W. Adson and J. R. Coffey[13] contributed an article, in which they pointed out that there is often a tense band in the scalenus anticus muscle closely applied to the nerve trunks, and division of this band gives just as good results as removal of the cervical rib. I have operated on seven occasions, and all were relieved of their pain and disability. None of them had a genuine cervical rib. Only in three was the costal process of the seventh cervical vertebra resected, in four part of the first rib was removed. In four cases the scalenus anticus was divided, as well as bone removed. In one case there was a bony spike, directed downwards, projecting from the tip of the costal process of the seventh cervical vertebra. From this spike a tense fibrous band stretched down to the first rib, and the lower cord of the brachial plexus passed over this band. It was divided. It is therefore clear that pain in the distribution of the ulnar nerve, with muscular wasting, may be caused by the pressure either of a tense fibrous band in a muscle, or of the first rib, and that it may be cured when one or other of these structures is divided. If so, it encourages us to seek for a similar cause and treatment in cases of sciatica.

It is true that my experience of operation is confined to three cases, but at the present time it is seldom that patients with sciatica are referred to a surgeon. On the other hand, if it proves true that a very simple operation, to divide a tense fibrous band, is sufficient to cure the sufferer, many patients would be glad to get rid of their sciatica in such an easy way.

Whether it is possible to distinguish between sciatica due to interstitial neuritis (if such a condition exists) and pressure-sciatica only further experience can decide. Neither tenderness of the nerve trunk on deep pressure nor Lasègue's sign are sufficient to prove that the symptoms are due to neuritis.

The fibrous band found and divided in my cases appears to be present quite often in subjects in the Anatomical Department. Professor Whitnall contributes the following description of the condition :—

"In the Anatomical Department of Bristol University eleven subjects were examined in the dissecting-room for the presence of this band. It was found as a definite formation on one or the other side in six, and on both sides in two of these. It lay exactly in the position that Mr. Short had found at operation, being felt as a tense crescentic edge of resilient tissue, seated as it were along the bony margin of the deepest part of the greater sciatic notch, and curving under and up forwards round the anterior border of the broad flat sciatic nerve which border, incidentally, comprises the lateral popliteal fibres, distributed to the outer side and front of the leg. Dissection showed it to be a thickened extension of the fascial covering of the gluteus minimus. So, since the lower hinder origin of this muscle generally extends as far as the anterior margin of the greater sciatic notch, such projection of its edge to form a band will lie beneath the emerging and superimposed sciatic nerve, and be in a position to be closely pressed upon by the latter in flexion of the thigh. At its upper forward end the band can be traced to pass over the edge of the pyriformis, where it is perforated by the superior gluteal vessels and nerve ; it then loses its identity by blending with the fascia covering the superficial aspect of pyriformis. There has not been found by us any previous description of this structure in the anatomical or clinical literature.

"From the anatomical dispositions of the parts concerned it is suggested that access to this particular region is best reached by 'spanning' the centre line of the upper third of the gluteus maximus. The exploring finger can then find the pyriformis ; beneath this it will feel the sciatic nerve. It must now be passed along the anterior forward (or lower in the prone position) edge of the nerve to seek a possible band ; for if passed medially or above it it may find the edge of the lesser sacro-sciatic ligament which has no direct relation to the nerve. Again, a blunt cut to release tension of the band should be directed downwards, in this prone position, in a direction towards the greater trochanter of the femur, so avoiding any possible implication of the superior gluteal vessels, which emerge from the pelvis above the pyriformis."

It should be borne in mind that if this gluteus minimus band presses on the nerve at all the external popliteal fibres will suffer most, and this accords with clinical experience ; wasting is most marked in the muscles supplied by that nerve. It might be thought that pressure from a band, growth or bone would not cause the sciatic nerve trunk to be tender, but according to Maclean[14] the tenderness is found both in pressure and in neuritis cases.

This method of operating for sciatica has been mentioned to other surgeons, and from personal communications I learn that they have had a number of successes. Probably the bands that Crawford Renton cut included the one here described.

It is difficult at present to define the exact indications for and limitations of the operation. It is not called for if the sciatica is due to fibrositis or pelvic growths, or spinal neoplasm, or in sacroiliac disease, or in cases with lumbar scoliosis. Nor is it called for until the simpler and generally recognized methods of treatment have been tried and failed. It begins to be indicated for the intractable cases in which no definite cause is found, and particularly when there is definite muscular wasting of the muscles supplied by the external popliteal nerve. It is not to be regarded as a cure-all ; it will only succeed in those cases of sciatica, probably a minority, in which the pain is due to the band pressing on the nerve, and in one of my three cases the operation was a partial failure.

### Summary.

Three cases are described in which sciatica was due to a fibrous band pressing on the nerve where it emerges from the sacrosciatic foramen. This band can often be demonstrated in the Anatomical Department. Division of the band may cure the sciatica.

REFERENCES.

[1] *Brit. Med. Jour.*, April, 1921, p. 557.

[2] *Proc. Royal Soc. Med.*, Nov., 1934, Section Phys. Med., p. 73.

[3] *Lancet*, 1927, ii. 53.

[4] *Journal Bone and Joint Surgery*, Jan., 1934, p. 126.

[5] *Jour. Amer. Med. Assoc.*, 102, 5th May, 1934, p. 1,465.

[6] *Irish Jour. of Med.*, Nov., 1931, p. 583.

[7] *Klin. Wochen*, 1927, vi. 739.

[8] *Acta. Medica Scandinavica*, x., 1925.

[9] *Scottish Med. and Surg. Jour.*, 1897, p. 35. *Proc. Roy. Soc. Med.*, Surgical Section, May, 1908, p. 167.

[10] *Brit. Med. Jour.*, April, 1921, p. 557.

[11] *Brit. Jour. Surg.*, Oct., 1919, p. 168.

[12] *Ann. Surg.*, June, 1927, p. 858.

[13] *Ann. Surg.*, June, 1927, p. 839.

[14] *Clin. Jour.*, May, 1931, p. 223.

## Figures and Tables

**Figure f1:**